# Smoking and multiple sclerosis: development and feasibility study of a MS-specific smoking cessation intervention

**DOI:** 10.1177/17562864251391057

**Published:** 2025-11-12

**Authors:** Alex M. Keller, Daniel Kotz, Claudia H. Marck, Alexander Wöhler, Christoph Heesen, Karin Riemann-Lorenz

**Affiliations:** Institute of Neuroimmunology and Multiple Sclerosis (INIMS), University Medical Centre Hamburg-Eppendorf, Martinistrasse 52, Hamburg 20246, Germany; Institute of General Practice, Addiction Research and Clinical Epidemiology Unit, Centre for Health and Society, Medical Faculty and University Hospital Düsseldorf, Heinrich Heine University Düsseldorf, Düsseldorf, Germany; Disability and Health Unit, the Melbourne School of Population and Global Health, The University of Melbourne, Melbourne, VIC, Australia; Institute of Neuroimmunology and Multiple Sclerosis, University Medical Centre Hamburg-Eppendorf, Hamburg, Germany; Institute of Neuroimmunology and Multiple Sclerosis, University Medical Centre Hamburg-Eppendorf, Hamburg, Germany; Institute of Neuroimmunology and Multiple Sclerosis, University Medical Centre Hamburg-Eppendorf, Hamburg, Germany

**Keywords:** behavior change, evaluation, intervention development, multiple sclerosis, smoking cessation

## Abstract

**Background:**

Tobacco smoking is an established risk factor for accelerated multiple sclerosis (MS) progression and worse MS symptoms. Generic smoking cessation programs might not fully meet the needs of people with MS (pwMS), as they don’t address MS-specific barriers influencing smoking behavior (e.g., worries about relapses when quitting). Yet, no MS-specific smoking cessation interventions have been evaluated.

**Objective:**

This study aimed to develop an MS-specific smoking cessation intervention.

**Design:**

This is an intervention development and initial feasibility study, informed by the Behavior Change Wheel and the design and evaluation framework for digital health interventions, which have been successfully utilized before, including in MS contexts.

**Method:**

Between January and December 2024, we developed MS-specific information videos to supplement an existing smoking cessation intervention. We used identified intervention functions and results from preceding studies to identify the most effective way to change smoking behavior in pwMS. For the evaluation of the videos, we developed a theory-based questionnaire, and recruited pwMS and MS experts via our MS day clinic for assessment. The evaluation informed final revised videos for integration into the existing program to form a MS-tailored smoking cessation intervention.

**Results:**

We identified five out of nine intervention functions from the behavior change wheel to be relevant and created six videos based on these functions. The content of the videos includes, among other things, education about the connection of smoking and MS, and persuasion and incentivization about the positive effects of quitting. Eleven pwMS and five MS experts assessed the material. Overall, the videos were perceived as understandable and appropriate in length in both groups. The modified smoking cessation intervention includes all videos, integrating them into a structure of five online-meetings across 3 weeks.

**Conclusion:**

The successful development of education videos using the Behavior Change Wheel, as well as the positive findings from our feasibility testing underline the potential of our video-based approach in the context of smoking cessation for pwMS. Next, the modified smoking cessation intervention should be tested for feasibility, acceptability, and efficacy. If successful, this approach could be implemented widely for people with MS.

## Plain language summary

Developing a program to help people with multiple sclerosis to quit smoking

People with multiple sclerosis (MS) who smoke often experience faster disease progression and worse symptoms. However, existing programs that help people quit smoking may not be well-suited to meet the unique needs of those living with MS. To address this, we developed a smoking cessation program specifically for people with MS. Our team created six short videos that explain how smoking affects MS and how quitting can help. These videos were reviewed by people with MS and MS experts. Both groups gave positive feedback. The final program includes the videos and takes place over five online sessions in three weeks. The goal is to support people with MS in quitting smoking using content tailored to their condition. The next step is to test how well this new program works and whether people find it helpful. If it proves effective, it could become a widely used tool to help people with MS stop smoking.

## Introduction

In Germany, approximately 280,000 people live with multiple sclerosis (MS),^1^ a chronic autoimmune disease likely caused by both genetic and environmental factors.^2^ MS affects the central nervous system and can therefore lead to a variety of symptoms. Most common are spasticity and coordination problems, fatigue, or cognitive deficits, such as attention or memory deficits.^3^ Smoking, both active and passive, is recognized as a significant risk factor for increased disease activity and faster progression in people with MS (pwMS).^4–6^ Additionally, pwMS who smoke have higher mortality rates,^7^ an increased risk for depression, anxiety and burden of T2-lesions,^8,9^ and lower quality of life^10^ compared to nonsmokers with MS. Importantly, smoking cessation has been shown to slow down disease progression and decrease the risk of reaching different disability milestones to a level comparable to people who never smoked.^11,12^ According to studies looking at MS populations, the prevalence of smoking among pwMS in Germany ranges from 19% to 24%,^9,13^ which is only slightly lower compared to the smoking prevalence in the general German population at 28%.^14^ The similarity of the prevalence rates and findings from interviews with pwMS^15^ both suggest that the MS-diagnosis alone is often not a sufficient motivating factor for successful smoking cessation.

Recent studies by colleagues in Australia as well as our group, have highlighted similar MS-specific motivators for continuing smoking, as well as MS-specific barriers to quitting.^15,16^ In one of the qualitative studies, we interviewed 15 pwMS and analyzed the data for MS-specific motivators and barriers influencing smoking behavior, as well as for their wishes and needs in terms of assistance.^15^ Shortly summarized, we found that pwMS had low knowledge about the connection of smoking and MS, that the MS diagnosis influenced their motivation to quit, and that they wished for better communication with MS experts as well as for more peer exchange in the context of smoking cessation. Collectively, our and the Australian studies suggest that generic smoking cessation interventions designed for the general population might not fully meet the needs for pwMS and could therefore be less effective compared to a tailored intervention. Generic programs, for instance, would not inform participants about MS-specific health-consequences that smoking can have, nor would they address concerns of pwMS who might fear that stress induced by nicotine withdrawal could trigger a relapse. Tailored interventions for different target groups have shown to be helpful in addressing specific barriers and needs and in promoting smoking cessation.^17^ In the context of MS, calls for more research have been made to assist smoking cessation among pwMS.^18,19^ Yet, no smoking cessation interventions have been tested in MS populations.^4^

We aim to fill this gap with this study. Its primary aim is to describe the development process of an MS-specific smoking cessation intervention. Subsequent phases will evaluate the intervention’s feasibility and efficacy.

## Methods

When reporting our intervention development, we followed the “Guidance for reporting intervention development studies in health research” (GUIDED),^20^ to increase transparency and potential reproducibility.

Our research is guided by the framework for developing and evaluating complex interventions, as proposed by the Medical Research Council (MRC).^21^ This framework emphasizes key aspects of intervention development, like understanding the context, developing and refining a clear programme theory, engaging stakeholders, addressing key uncertainties, and considering economic factors across all phases of research. By following this framework, we ensure that our intervention is robust, adaptable, and applicable in real-world settings.

**Figure 1.**
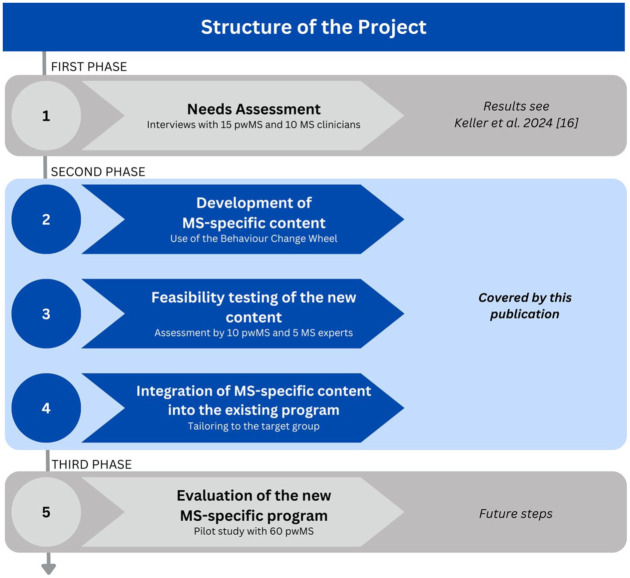
Structure of the overarching project. MS, multiple sclerosis; pwMS, people with multiple sclerosis.

This study represents the second out of three phases of a larger project, which is described in Figure 1. The main aim of this project is to develop and evaluate an online and MS-specific smoking cessation program for pwMS.

In the first phase, we assessed the needs and wishes from pwMS and MS clinicians regarding support for smoking cessation. The results have already been published^15^ and will be briefly summarized in the Results section, as they formed the basis for the second phase.

The second phase—which is the subject of this article—comprises of three steps. In the first step, we developed MS-specific content in the form of short educational and information videos to use for the online smoking cessation intervention. In a second step, we tested the feasibility of these videos with pwMS and MS experts. In a third step, we integrated the videos into an existing generic online smoking cessation program to create an adjusted and therefore, MS-specific smoking cessation intervention for pwMS.

In the third phase, we will evaluate the adjusted program with approximately 60 pwMS. The study protocol for that part of the project has been made available online.^22^

Here, we will elaborate on the methodology of the three steps of the second phase.

### Step 1: Development of MS-specific content

For the development of the educational and information videos, we used the behavior change wheel (BCW).^23^ The BCW provides a stepwise and easy-to-follow approach to design behavior change interventions and proved to be a useful tool to guide the development of the MS-specific videos. It synthesizes 19 different frameworks of behavior change into one coherent framework and is commonly used in various contexts, including the development of different smoking cessation interventions,^24,25^ or for physical activity interventions for pwMS.^26^

The first part of the BCW-process is about understanding the problematic behavior, by defining it and identifying what needs to change. For that purpose, the COM-B model is used. According to that model, for a behavior to change or occur, a person needs to have the capability (physical and psychological), opportunity (physical and social), and motivation (reflective and automatic).^27^ We assessed the factors influencing smoking behavior of pwMS in the first phase of our study and mapped them onto the COM-B model.^15^

The next part of the BCW is about identifying intervention functions (IF). IFs are broad categories “by which an intervention can change behaviour” (20, page 109). A list of all IFs and their definitions can be found in Table 1.

**Table 1.** Intervention functions and definitions according to Michie et al.^23^.

Intervention function	Definition
Education	Increase knowledge or understanding
Persuasion	Induce positive or negative feelings or stimulate action
Incentivization	Create an expectation of reward
Coercion	Create an expectation of cost
Training	Imparting skills
Restriction	Using rules to reduce the opportunity to engage in the target behavior
Environmental restructuring	Changing the physical or social context
Modelling	Providing an example for people to aspire to
Enablement	Increasing means/reducing barriers to increase capability or opportunity

Each IF can be connected with one or more components of the COM-B model. Using the BCW, we connected the identified factors of the COM-B model from the first phase of our study with the IFs. Each possible connection can be found in Table 2. We then used the APEASE criteria (affordability, practicability, effectiveness, acceptability, safety, equity) to identify the most relevant IFs within our context. The identified IFs were then used to guide the further development of the videos.

**Table 2.** Connection of COM-B components and their respective intervention functions based on Michie et al.^23^

COM-B component	Intervention function
Physical capability	Training
Psychological capability	Education, training, environmental restructuring, enablement, modelling
Reflective motivation	Education, persuasion, modelling, enablement, incentivization, coercion
Automatic motivation	Training, incentivization, coercion, environmental restructuring, persuasion, modelling, enablement
Physical opportunity	Training, restriction, environmental restructuring, enablement
Social opportunity	Restriction, environmental restructuring, modelling, enablement

COM-B, Capability, Opportunity, Motivation, Behavior.

Next, our team of researchers from the fields of health sciences, psychology, and medicine discussed and decided how the IFs can be utilized in the conceptualization of the educational and information videos about smoking and MS, to fulfil the needs and wishes of pwMS identified in phase I of the study. Scripts and ideas for six different videos were drafted and discussed within the team of researchers and with a professional graphical designer, specialized in the field of science illustration. Storyboards for each video were drafted and together with the scripts discussed within our research team and revised if deemed necessary. Three of these videos were fully illustrated and animated, while one was designed as an explainer video featuring an MS expert, complemented by graphical overlays. The MS expert was recruited from our MS day clinic, and the video was recorded at our facility after written consent was provided. For the expert, we looked for someone with several years of experience in the field of MS, while other characteristics (e.g., age, gender) were irrelevant in selecting an appropriate expert. For two further videos, we invited a person with MS who quit smoking after the diagnosis and recorded a semistructured interview with them about their experiences before, during, and after they had stopped smoking. We specifically looked for and chose a pwMS, who had stopped smoking after they had received their diagnosis, while other characteristics (e.g., age, gender) were irrelevant in selecting an appropriate participant. We recruited that person via the MS day clinic from our institution by approaching them in person, and recorded the interview in a room in our facility, after we received written consent. The interview was then cut into two videos, each dealing with a separate topic of the process of smoking cessation. The final versions of the videos were sent to the participant to provide an opportunity for comments or feedback; however, no requests for changes were made.

### Step 2: Feasibility testing of the new content

To test the newly developed videos for feasibility, we used the design and evaluation framework for digital health interventions, as proposed by Kowatsch et al.^28^ The framework’s objective is to assist the conceptualization and implementation of digital health interventions (DHIs). This framework fits our context, as our smoking cessation program will be an online intervention with integrated digital material like our videos. Kowatch et al. synthesized a system with 13 categories of evaluation criteria for DHIs. Each criterion defines an important aspect of an intervention and gives indications on how to evaluate it. We identified five of these categories to be relevant in our context: Ease of use (required effort), content quality (accuracy, relevancy, consistency), perceived benefit (believe, that DHI helps), perceived enjoyment (engagement), and aesthetics (design, color, fonts). We used the identified categories to create a self-developed feasibility questionnaire. A draft of the questionnaire was discussed within our research team. After small adjustments, a version with 15 statements was finalized. Exemplary statements are “I found it easy to understand the content presented in the video” (Ease of use), or “The content of the video is relevant to people with MS who smoke” (Content quality). Each statement from the evaluation questionnaire could be answered on a four-point-Likert-scale, ranging from *Strongly Disagree* with a score value of 1, to *Strongly Agree* with a score value of 4. Due to the formulation of the statements, higher scores mean a more positive feedback. The questionnaire also contained four open questions, like, for example, “What did you like most about the video?” (Perceived Enjoyment), and one question, where participants were asked to give each video a summarizing grade, according to a grading system from 1 (Very good) to 6 (insufficient), which is commonly used in Germany. The questionnaire and corresponding categories of evaluation criteria can be found in the Supplementary Material. Although we did not pilot test our self-developed questionnaire, our research team, which has applied the framework of Kowatsch^28^ to design and implement questionnaires in various prior studies,^29,30^ revisited the questionnaire after its first use. There, it was agreed that no changes were necessary and its use could be continued.

#### Participants and setting

For evaluation, we presented the videos to pwMS and to MS experts (neurologists, MS nurses). Participants were eligible if they (a) self-reported a MS diagnosis and (b) were current or former smokers. For MS experts, inclusion required a professional background and experience in the field of multiple sclerosis. For both groups, there were no additional exclusion criteria. Recruitment took place at the MS day clinic of our institution at the University Medical Centre Hamburg-Eppendorf. Participants were either approached in person, via phone calls or emails. After providing informed consent, all participants were asked to have a look at the videos and fill out one questionnaire for each video. This was done either in rooms at our facility or at the homes of the participants. In total, 16 participants were included. Each video was assessed by at least five pwMS and five MS-experts.

#### Statistical methods

Participants’ answers were reported descriptively only, using a heat map. In this context, a heat map allowed us to visualize individual responses, while also enabling intuitive comparisons both between and within our given groups (pwMS and MS experts) for each video and each item. Means were reported where appropriate. We had no missing data. The results from pwMS and MS experts were reported separately. The answers from the open questions were summarized and presented narratively. Analysis and plotting were done with the software Excel.

### Step 3: Integrating the new content into the basic program

We integrated the six evaluated and MS-specific videos and their content into an existing smoking cessation program designed for the general population: the so-called Rauchfrei-Programm (Smoke-Free Program),^31^ which was developed by the Institute for Therapy Research (IFT, Institut für Therapieforschung). This *basic* program comprises four online group meetings and is based on principles of cognitive behavioral therapy as well as motivational behavior change techniques. It undergoes frequent quality insurance checks and evaluation. The online group meetings are led by certified trainers from the IFT, who must have a background in psychotherapy, health- or social sciences.

In collaboration with a psychologist from the IFT, and an experienced smoke-free trainer, our interdisciplinary team of health scientists, neurologists, and psychologists discussed potential adaptations to the *basic* program to incorporate the videos for an *adjusted* MS-specific program. From March to December 2024, regular meetings were held to systematically review and adjust the structure of the *adjusted* program. In each meeting, suggestions were proposed from all sides, discussed, and modified as necessary to accommodate the new content while maintaining the core elements of the established program. Ultimately, we agreed on a revised program structure, now consisting of five instead of four group meetings with the MS-specific content integrated throughout the sessions. Results were collected in a comprehensive Trainer Manual, which was developed to outline the revised structure of the program and to ensure consistent delivery across different trainers.

## Results

### Development of MS-specific content

**Figure 2.**
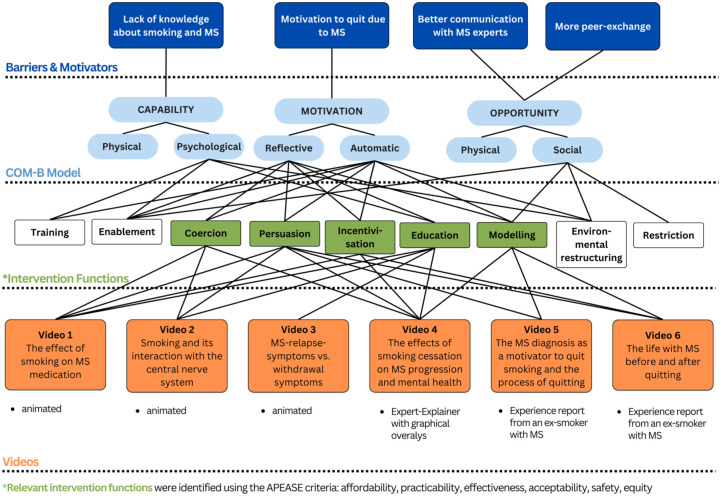
The process of the development of MS-specific information videos, based on the Behavior Change Wheel. MS, multiple sclerosis.

The development of the videos was based on the findings from the first phase of our study (see Figure 1), where we identified barriers, motivators, and needs of pwMS in the context of smoking cessation. We shortly summarized our findings in the Introduction. Now, Figure 2 showcases the process of how we got from these results to the development of six educational and information videos. We mapped the findings from the first phase of our project (dark blue) onto the COM-B model (light blue), connected its components with intervention functions according to the BCW (green), and utilized the intervention functions in the conceptualization of six videos (orange).

The first identified barrier influencing smoking behavior of pwMS was their lack of knowledge about how smoking impacts on MS. This describes a form of psychological capability (COM-B), which can be defined as knowledge or psychological skill, to deal with the topic of smoking and MS. This capability can be influenced by education (intervention function). The IF of education is defined as the increase of knowledge or understanding of a topic. In our case, this is achieved through videos 1–4, which address the following topics: (1) the effects of smoking on MS medication, (2) the interaction between smoking and the central nervous system, (3) a comparison of MS relapses and withdrawal symptoms, and (4) the impact of smoking cessation on MS progression and mental health.

The most relevant identified motivator influencing smoking behavior of pwMS was their gained motivation to quit, due to their MS diagnosis. This describes both reflective and automatic motivation (COM-B). Reflective motivation can be the intend to stop smoking, while automatic motivation can be the impact of anticipated benefits, that smoking cessation might have on the MS. This motivation can be increased, for example, with the transfer of knowledge (IF education), with influencing the expectation of disease worsening (IF coercion), with influencing the expectation of better health outcomes when quitting (IF incentivization and persuasion), or with providing an experience report of a successful quitter with MS (IF modelling). We used these IFs to increase motivation in the conceptualization of all six videos, as shown in Figure 2. The content of videos one to four were already explained above, but videos five and six are both experience reports from an ex-smoker with MS, who talks about (5) their MS diagnosis as a motivator to quit smoking and the process of quitting, and about (6) the comparison of their life with MS before and after they quit smoking.

Finally, the identified wish for better communication with MS experts and more peer exchange was accounted for with targeting social opportunity (COM-B). Social opportunities can be interpersonal factors, that influence how one thinks about the topic of smoking in relation to their MS. With our videos five and six, we utilized the IF of modelling to influence the social opportunity of pwMS. Modelling describes a positive example of a person, like a person with MS who successfully quit smoking, for others to aspire to. Also, video four, which is an MS expert explaining the effects of smoking cessation on MS, is used to influence the social opportunity of our target group.

Summarized, each of our six videos uses a combination of different intervention functions to influence the capability, opportunity, and motivation of people with MS, which, as an outcome, influence the smoking behavior of pwMS.

## Assessment of the digital information material

In total, we invited 16 participants to assess our videos. Eleven were pwMS and five were MS-experts. Among pwMS, five were female and six male, with a median age of 53.3 years. All five experts were female, with a median age of 32 years, and an average of 12 years working experience with MS. All main characteristics of our sample can be found in Table 3.

**Table 3.** Sample characteristics.

	pwMS (*n* = 11)	MS-experts (*n* = 5)
Gender		
Female	5	5
Male	6	0
Median age (years)	53.5	32.0
Smoking status		
Current smokers	6	0
Former smokers	5	3
Never-smokers	0	2
Education		
Lower secondary education	1	0
Upper secondary education	2	0
Vocational training	1	3
University entrance qual.	3	0
Tertiary education	4	2
MS-subtype		
Relapsing remitting	5	
Primary progressive	3	n/a
Secondary progressive	1	
Unclear	2	
Working Experience with MS (average in years)	n/a	12
Median Time since diagnosis (in years)	4.5	n/a

MS, Multiple Sclerosis; n/a, not applicable; pwMS, people with Multiple Sclerosis; qual., qualification.

**Figure 3.**
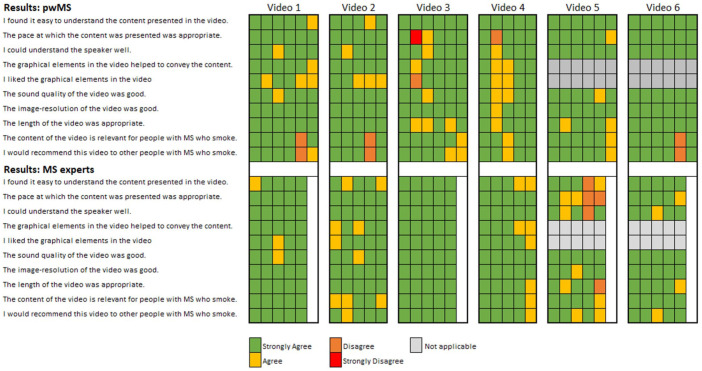
Heat-Map of the assessment of the digital information material by pwMS (top) and MS experts (bottom). Each column represents the answers for each item of one individual participant. Each video was therefore assessed by five MS experts and five or six pwMS. MS, multiple sclerosis; pwMS, people with Multiple Sclerosis.

Figure 3 presents a heat map summarizing participants’ feedback, with colors corresponding to Likert-scale responses (e.g., Green = Strongly Agree = Score of 4; Red = Strongly Disagree = Score of 1). Overall, all videos received positive feedback, defined as an average score greater than 3. No item scored below 3.4 across all videos and participant groups. A table with mean scores for each video and each item can be found in the Supplemental Table.

A key item assessing the *ease of use* states: “I found it easy to understand the content presented in the video.” Here, mean scores ranged from 3.4 (standard deviation (SD) = 0.9) to 4 (SD = 0) across all videos. The heat map reveals that MS experts provided slightly lower scores than pwMS, as indicated by a higher occurrence of yellow and orange cells.

Another key item evaluating *content quality* states: “The content of the video is relevant for people with MS who smoke.” Here again, mean scores fell between 3.4 (SD = 0.5) and 4 (SD = 0). However, pwMS rated three videos lower than MS experts.

A third key item assessing the *perceived benefit* of the videos states: “I would recommend this video to other people with MS who smoke,” which yielded mean scores between 3.5 (SD = 0.8) and 4 (SD = 0). Here, pwMS assigned lower ratings to four videos compared to MS experts.

Notably, Video 3 and Video 5 demonstrated the most pronounced differences between the two groups. Video 3 was rated more favorably by MS experts, while pwMS provided more critical feedback across multiple items. In contrast, Video 5 was better received by pwMS, whereas MS experts were more critical, as reflected in the heat map.

### Open questions

The feedback on the open questions highlighted several aspects. A concise and clear explanation of the content was frequently mentioned as a positive aspect, as it was described by our participants to convey information in an accessible manner while maintaining a neutral and nonjudgmental tone. The focus on positive outcomes, including life improvements following smoking cessation, was regarded as encouraging and the experience report from a former smoker was perceived as authentic.

On the other hand, participants expressed that the focus on specific treatments or symptoms, like MS relapses, made the content less relatable for those with other MS subtypes or treatments. Another criticism was that sometimes the video’s illustrations were inconsistent with the verbal explanations.

In terms of suggestions for improvement, a common recommendation was to broaden the scope of the video to make them more relatable for people with all different forms of MS, and to address various medications or treatments. Even if there are no specific studies available, participants suggested acknowledging other treatments and their potential interactions with smoking.

### Revisions

As a result from the gathered feedback from our participants, minor revisions were made to our videos. First, some of the animated videos received small graphical adjustments to improve the clarity at points, where some participants indicated inconsistencies with the verbal explanations. Second, we shortened video five and six and added chapter headlines between cuts, to make them easier to follow and to avoid redundancies. Finally, we added a short section to the video about the effects of smoking on MS medication, improving the explanation about the lack of studies about the impact of smoking on other MS medications than the ones already mentioned in the video. The revised videos were reviewed internally by our research group, but not by new participants like before.

### Integrating the new content into the basic program

**Figure 4.**
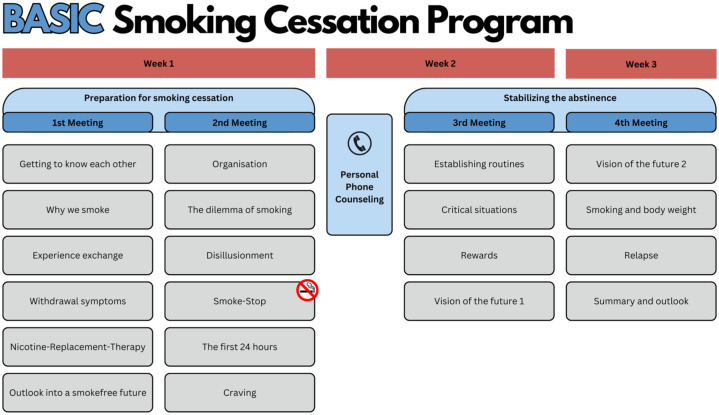
Structure of the basic smoking cessation program before MS-specific adaptations. MS, multiple sclerosis.

In a third and final step, we integrated the MS-specific content into the already existing basic smoking cessation program. The structure of the original basic program is shown in Figure 4. It consists of four meetings of around 90 min during a time span of around 3 weeks. Each meetings consists of various modules, which deal with different aspects of smoking cessation. The first two meetings are preparing the participants for their smoking cessation, while the last two meetings aim to stabilize the abstinence. Between meeting two and three, each participants gets a 10-min personal phone counselling with the trainer, to talk about individual issues and problems regarding the smoking cessation.

**Figure 5.**
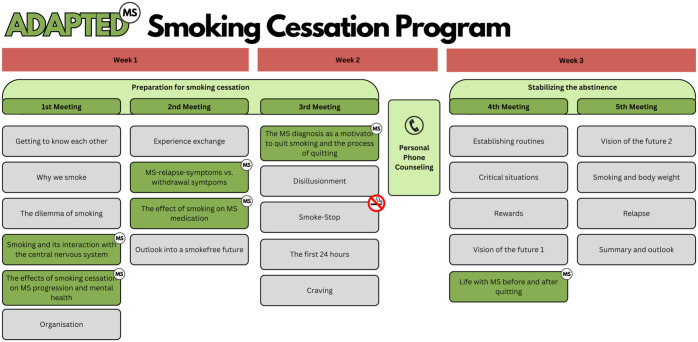
Structure of the adapted smoking cessation program with integrated MS-specific content. Green boxes with the “MS”,-Icons indicate the added MS-specific content. MS, multiple sclerosis.

Figure 5 showcases the structure of the adapted and MS-specific smoking cessation program. Here, the MS-specific content is highlighted with the green modules. In order to not exceed the 90 min per meeting, it was decided to add a fifth meeting to the overall structure, and to accommodate the new content without removing established content from the basic program. The established content is regularly evaluated for quality by the IFT and constitutes an integral part of their proven smoking cessation approach. Our aim was therefore to preserve the original structure as much as possible, while integrating the MS-specific elements in a coherent way. Now, the first three meetings prepare for the smoking cessation, while the last two meetings still aim to stabilize the abstinence. The personal phone counselling in the adapted version is between meeting three and four.

## Discussion

In this study, we developed and evaluated educational and information videos and integrated them into a MS-specific smoking cessation intervention. The behavior change wheel proofed to be a useful and easy-to-use tool in the conceptualization and development of these videos, specifically the COM-B model and the layer of intervention functions. They provided a systematic approach to identifying behavioral determinants and linking them to appropriate intervention strategies. Another study within the MS context also used the BCW to develop a physical activity intervention for pwMS.^26^ Different to us, they applied the framework more comprehensively, including mapping of behavior change techniques and policy categories. While the behavioral targets and contexts of their intervention differ from ours, the different approaches to the BCW from both their and our study highlight the flexibility and usefulness of the BCW in developing health interventions adapted to the needs of pwMS.

The results from the feasibility testing show that the videos were rated very positively. These findings underline the potential of digital approaches in the context of smoking cessation for pwMS, since there are—to our knowledge—no other digital smoking cessation programs out there which have been specifically developed for pwMS or people with other neurological or autoimmune conditions. Different studies of varying settings, however, point at a high acceptability and positive effects of educational videos for smoking cessation interventions,^32^ including smoking cessation interventions for people with chronic illnesses.^33^ Generally, digital forms of interventions are increasingly used in health care settings^34^ and can have positive effects on smoking cessation.^35,36^ In MS populations, various digital health interventions have already shown to be effective.^37,38^ It is argued that pwMS are especially suited for digital interventions, as many are relatively young and digitally literate on average compared to patients with other chronic illnesses.^38^ In addition, digital formats like our videos can be a helpful method to increase self-paced learning and motivation^39^—features that may be beneficial for pwMS who experience fatigue or cognitive limitations. The successful development and positive evaluation of our videos suggest that digital information material like these videos can be a useful tool in a smoking cessation intervention designed specifically for pwMS as well. Furthermore, the benefits of a digital approach to health interventions can not only be beneficial to the recipients but also to the interventionists.^40^ For instance, it allows for a flexible approach in the implementation, as shown within this study by the adaptation of the existing smoke-free program, and it will make it easier to scale the intervention to a broader target group and improve its access. Working with videos therefore offers a flexible and scalable approach for more content development. This format makes it feasible to create additional modules tailored to the needs of different MS subtypes. For example, participant feedback suggested that expanding the content to reflect the experiences or needs of people with progressive forms of MS could help ensure that individuals across the entire MS spectrum feel adequately represented. This became particularly apparent in responses to Video 3, which discussed the difference between relapse and nicotine withdrawal. While MS experts rated the video positively, pwMS were more critical—possibly because those with progressive forms of MS felt less addressed by content focused on relapses.

### Strengths and limitations

For the first time to our knowledge, digital smoking cessation information material has been developed and evaluated specifically for MS patients. For our feasibility testing we included both pwMS and MS experts for a broad insight into the perception of these two relevant groups. However, the overall sample size was small, and persons with a higher interest and affinity for digitalization might have been more likely to participate and more likely to assess the videos positively. If the intervention indicates effectivity, subsequent work should strive to reduce potential participation bias—for example, by employing more recruitment strategies. However, we acknowledge that actively reaching out to people, for example, with MS with limited digital access or confidence, or integrating dedicated digital support to support them is a significant challenge that requires specific and dedicated effort in future research. Furthermore, participants might provide socially desirable responses when asked about feasibility and acceptability, which we aimed to minimize by enabling participants to answer anonymously. We did not pretest our self-developed questionnaire in a separate pilot sample, but it underwent an internal review process within our research team instead. This may have limited our ability to detect possible comprehension or interpretation problems. However, we judge the impact of this measurement bias on our findings to be low, given the focused scope of the questionnaire and the overall consistency of responses observed. While participants across all educational levels unanimously found the content of the videos easy to understand, future studies could consider intentionally including more individuals with low literacy or cognitive issues. One potential strength of short and comprehensive videos is their accessibility for these groups, which could help mitigate the risk of increasing health inequities if such interventions are primarily effective only for individuals with higher educational levels. Accessibility could further be increased by the integration of subtitles (even in different languages), or by providing transcripts and Supplementary Material such as handouts with more explanations or background information.

With the behavior change wheel, we used an established framework for the development and conceptualization of behavior change interventions, which have been used in previous applications in smoking cessation (24,25). Using this framework ensured a systematic and theory-driven development of our video content. This strengthens the conceptual rigor of our intervention and enhances its reproducibility. Further, by primarily using videos to convey MS-specific information within the program, the approach aims to relieve the trainers conducting the courses of the need for extensive subject matter expertise, which will be helpful, when aiming to implement the MS-specific intervention into basic care.

## Conclusion

In summary, the results of this study show that educational and information videos can be a promising component of an MS-specific smoking cessation program, based on the feedback of both pwMS and MS experts. As described in Figure 1, we will pilot the adjusted MS-specific smoking cessation intervention with the integrated videos with approximately 60 pwMS in the next and third phase of our project. There, we will test the intervention for feasibility and acceptability, as well as limited efficacy as assessed by smoking cessation rates. In a future phase, it will be also helpful to gather feedback from program trainers on the practicality of delivering the intervention with the integrated videos, to support successful implementation in routine care. If the pilot demonstrates positive outcomes, a randomized controlled trial can be planned, to test the intervention’s ability to reach our overarching goal, which is to increase smoking cessation rates among the population of pwMS. If successful, further research could examine how digital material like our videos and the overall described process of the intervention development could be adapted to different target groups as well, such as people with different neurological or chronic conditions, who might have specific smoking cessation needs as well.

## Supplemental Material

sj-docx-1-tan-10.1177_17562864251391057 – Supplemental material for Smoking and multiple sclerosis: development and feasibility study of a MS-specific smoking cessation intervention Supplemental material, sj-docx-1-tan-10.1177_17562864251391057 for Smoking and multiple sclerosis: development and feasibility study of a MS-specific smoking cessation intervention by Alex M. Keller, Daniel Kotz, Claudia H. Marck, Alexander Wöhler, Christoph Heesen and Karin Riemann-Lorenz in Therapeutic Advances in Neurological Disorders

## Declarations

**Ethics approval and consent to participate:** This research project was approved by the German ethics committee of the Hamburg Chamber of Physicians (registration number 2022-100779-BO-ff). Written informed consent was obtained from all participants involved in the study.

**Consent for publication:** Not applicable.

**Availability of data and materials:** The datasets used and analysed during the current study are available from the corresponding author on reasonable request.
