# Supplementary material for: Smoking and multiple sclerosis: development and feasibility study of a MS-specific smoking cessation intervention
Source: Ther Adv Neurol Disord. 2025 Nov 12;18:17562864251391057. doi: 10.1177/17562864251391057 (PMC12615918; doi:10.1177/17562864251391057)
Supplement: sj-docx-1-tan-10.1177_17562864251391057 – Supplemental material for Smoking and multiple sclerosis: development and feasibility study of a MS-specific smoking cessation intervention [file sj-docx-1-tan-10.1177_17562864251391057.docx]

Supplement Table: Mean results from the evaluation questionnaire (videos)

| Mean results from the evaluation questionnaire | Video 1 | | Video 2 | | Video 3 | | Video 4 | | Video 5 | | Video 6 | |
| --- | --- | --- | --- | --- | --- | --- | --- | --- | --- | --- | --- | --- |
|  | pwMS (n=6) | MS-experts (n=5) | pwMS (n=5) | MS-experts (n=5) | pwMS (n=6) | MS-experts (n=5) | pwMS (n=5) | MS-experts (n=5) | pwMS (n=6) | MS-experts (n=5) | pwMS (n=6) | MS-experts (n=5) |
| I found it easy to understand the content presented in the video. | 3,8 | 3,8 | 3,8 | 3,6 | 4,0 | 4 | 4,0 | 3,6 | 4,0 | 3,4 | 4,0 | 4 |
| The pace at which the content was presented was appropriate. | 4,0 | 4 | 4,0 | 4 | 3,3 | 4 | 3,6 | 4 | 2,8 | 3,8 | 4,0 | 3,8 |
| I could understand the speaker well. | 3,8 | 4 | 3,8 | 4 | 3,8 | 4 | 3,8 | 4 | 4,0 | 3,4 | 4,0 | 3,8 |
| The graphical representations in the video helped to convey the content. | 3,8 | 4 | 4,0 | 3,6 | 3,8 | 4 | 3,6 | 3,6 | n/a | n/a | n/a | n/a |
| I liked the graphic representations in the video. | 3,5 | 3,8 | 3,4 | 3,8 | 3,7 | 4 | 3,6 | 3,8 | n/a | n/a | n/a | n/a |
| The sound quality of the video was good. | 3,8 | 3,8 | 4,0 | 3,8 | 3,8 | 4 | 3,6 | 4 | 3,8 | 4 | 4,0 | 4 |
| The image-resolution of the video was good. | 4,0 | 4 | 4,0 | 4 | 4,0 | 4 | 3,8 | 4 | 4,0 | 3,8 | 4,0 | 4 |
| The length of the video was appropriate. | 4,0 | 4 | 4,0 | 4 | 3,5 | 4 | 3,8 | 3,8 | 3,7 | 3,4 | 4,0 | 3,8 |
| The content of the video is relevant for people with MS who smoke. | 3,7 | 4 | 3,6 | 3,4 | 3,8 | 4 | 3,8 | 3,8 | 3,8 | 3,8 | 3,7 | 4 |
| I would recommend this video to other people with MS who smoke. | 3,5 | 4 | 3,6 | 3,8 | 3,7 | 4 | 3,8 | 3,8 | 3,8 | 3,6 | 3,7 | 3,8 |
| Questions were answered using a 4-Point-Likert-Scale: 1 = Strongly Disagree to 4 = Strongly Agree; Results are shown in Means; Key Items highlighted in green | | | | | | | | | | | | |
